# Demographic characteristics and clinical-radiological correlation in patients with indications for Total Knee Arthroplasty: A cross-sectional study

**DOI:** 10.1016/j.clinsp.2024.100503

**Published:** 2024-10-01

**Authors:** Diego Ubrig Munhoz, Andre Giardino Moreira da Silva, Pedro Nogueira Giglio, Camilo Partezani Helito, Riccardo Gomes Gobbi, Luís Eduardo Passarelli Tirico

**Affiliations:** a Grupo de Joelho, Instituto de Ortopedia e Traumatologia, Hospital das Clínicas HCFMUSP, Faculdade de Medicina, Universidade de São Paulo, São Paulo, SP, Brazil; b Hospital Sírio Libanês, São Paulo, SP, Brazil; c HCor Hospital do Coração, São Paulo, SP, Brazil

**Keywords:** Total Knee Arthroplasty, Gonarthrosis, KSS, KOOS, Kellgren-Lawrence, Osteoarthritis

## Abstract

**Objective:**

The objective of this study is to correlate clinical symptoms and functionality, using the KOOS (Knee Injury and Osteoarthritis Outcome Score) and KSS (Knee Society Score System) scores, with the radiographic changes, using the Kellgren-Lawrence classification, in patients with knee osteoarthritis and indications for Total Knee Arthroplasty (TKA).

**Methods:**

120 patients (189 knees) with gonarthrosis and indications for TKA were included in the study. Demographic questionnaires were applied, and clinical and functional assessment was carried out using the KOOS and KSS scores. Knee radiographs were taken and graded according to Kellgren-Lawrence. The clinical scores were compared with the radiographic classification to establish a correlation between these two measurements. Statistical analysis was performed using the τ-Kendall correlation test.

**Results:**

Weak and inversely proportional correlations were found between the clinical scores and the radiographic classification. Among clinical scores evaluated, KSS showed the highest correlation with Kellgren-Lawrence classification (τ = -0.356; p < 0.001), followed by KOOS-quality of life (τ = -0.176; p = 0.004), KOOS-total score (τ = -0.166; p = 0.004), KOOS-function in daily living (τ = -0.160; p = 0.005) and KOOS-symptoms (τ = -0.159; p = 0.006). KOOS-pain (τ = -0.149; p = 0.01) and KOOS-sport and recreation function (τ = -0.142; p = 0.025) scores had the weakest correlations.

**Conclusion:**

There is a weak correlation between the clinical-functional scores of TKA candidates and their radiographic classification by Kellgren-Lawrence. Among clinical scores evaluated, KSS had the strongest negative correlation with the radiographic classification.

## Highlights

• Knee osteoarthritis is a common joint disease.
• Total Knee Arthroplasty (TKA) is the gold standard surgical treatment after failed conservative management.
• There is a weak correlation between radiographic worsening and functional impairment in patients with knee osteoarthritis and indication for TKA.

## Introduction

Osteoarthritis is the most common joint disease,^1^ and the knee is the most affected site,^2^ occurring in around 10% of the population over 60 years of age.^3^ The signs and symptoms include knee pain, stiffness and loss of range of motion, crepitus, loss of function, and need for orthopedic devices.^4^ Although the main complaint of patients is pain,^5^ it is not a reliable indicator of the severity of gonarthrosis when assessed in isolation, as patients adjust their activity levels to manage this symptom.^6^ Regarding the clinical assessment of patients with gonarthrosis, there are validated functional scores, such as KOOS (Knee injury and Osteoarthritis Outcome Score)^7^ and KSS (Knee Society Score System).^8^ Concerning radiographic assessment, Kellgren-Lawrence classification,^9^ the most widely used system, evaluates the presence of the classic signs of osteoarthrosis (narrowing of the joint space, presence of osteophytes, subchondral sclerosis, presence of deformity of the joint surface).

Previous studies have demonstrated a clinical-radiographic dissociation in patients with knee osteoarthritis. A population study that evaluated the radiographic prevalence of knee osteoarthritis (considering radiographic osteoarthritis patients with Kellgren-Lawrence ≥ 1), found up to 60% of asymptomatic patients among those with radiographic osteoarthritis.^10^ On the other hand, in the initial stages of osteoarthrosis, there is a symptomatic phase that precedes the radiographic changes.^11^, ^12^, ^13^ At this stage, magnetic ressonance imaging (MRI) may reveal lesions in the articular cartilage, subchondral bone, bone marrow, and/or menisci, which are likely associated with symptoms.^12^^,^^13^ Case et al., in their case-control study, showed that patients who developed radiographic osteoarthritis presented progressive symptoms of knee pain, stiffness and difficulties with functional tasks in the pre-radiographic phase.^13^ Therefore, there are patients with radiographic osteoarthritis with no symptoms, and vice versa.

In subjects with indications for Total Knee Arthroplasty (TKA), the gold standard surgical treatment for knee osteoarthritis, the Kellgren-Lawrence classification may underestimate the severity of gonarthrosis. In their study, Abdelaziz et al. demonstrated a discrepancy of 66.1% between the preoperative Kellgren-Lawrence classification and the intraoperative findings of joint degeneration in patients undergoing TKA.^14^ Therefore, a full clinical assessment and a better understanding of the correlation between symptoms and radiographic changes are essential to determine the right moment to recommend surgery.

Although it is well established in the literature that there is a weak correlation between clinical symptoms and radiographic changes in patients with knee osteoarthritis,^6^^,^^15^, ^16^, ^17^ the strength of this correlation can be variable in different phases of the disease. To date, there are still no studies that have evaluated the correlation between Kellgren-Lawrence classification and clinical-functional scores in TKA candidates, whose clinical and radiological parameters and their correlation are fundamental in therapeutic decision-making. In this context, this study aims to evaluate the correlation between KOOS and KSS clinical scores and the radiographic findings classified according to Kellgren-Lawrence in TKA candidates in a tertiary hospital. The secondary objective is to carry out an epidemiological analysis of the studied population, evaluating the variables of age, gender, Body Mass Index (BMI), comorbidities, and laterality of the joint disease.

## Methods

A cross-sectional study was performed in a tertiary public hospital following STROBE guidelines. The study was approved by the University of São Paulo Institutional Review Board with internal number CAAE 08496919.5.0000.0068, and an Informed Consent Form was signed by all participants.

A total of 328 patients with knee osteoarthritis and indications for TKA were selected. 118 had outdated registration data or contact was not possible, 52 did not agree to participate in the study, 20 did not turn up to the appointment, 10 had already undergone surgery and 8 had died. In total, 120 patients (189 knees) were included in the present study.

The inclusion criteria were patients with knee osteoarthritis (unilateral or bilateral) who had any indication for TKA between January 2007 and April 2018. Non-inclusion criteria included patients who did not respond to contact attempts, patients who had already undergone TKA and patients who refused to participate.

Demographic data collection was conducted using questionnaires, which included age, gender, weight, height, Body Mass Index (BMI), clinical comorbidities, and laterality of gonarthrosis. The analysis of functionality, quality of life, and physical parameters was carried out using two clinical-functional scores: KOOS (Knee Injury and Osteoarthritis Outcome Score) ‒ a clinical scale with 5 subsections ranging from 0 (worst situation) to 100 (best situation);^7^ and KSS (Knee Society Score System) ‒ a clinical scale, ranging from 0 (worst situation) to 100 (best situation).^8^

**Fig. 1:**
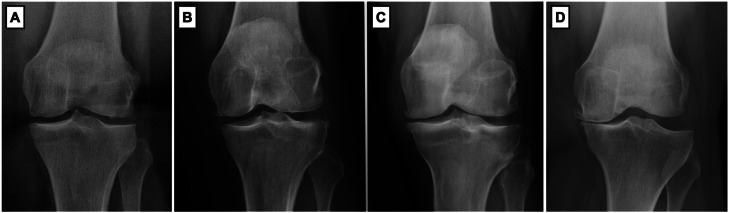
Knee radiographs classified by Kellgren-Lawrence system. (A) Kellgren-Lawrence 1 (doubtful joint space narrowing and possible osteophytic lipping); (B) Kellgren-Lawrence 2 (definite osteophytes and possible joint space narrowing); (C) Kellgren-Lawrence 3 (moderate multiple osteophytes, definite narrowing of joint space, some sclerosis and possible deformity of bone ends); (D) Kellgren-Lawrence 4 (large osteophytes, marked narrowing of joint space, severe sclerosis, and definite deformity of bone ends).

Radiographic analysis was carried out based on full-limb and knee radiographs, including an anteroposterior weight-bearing view, a lateral view with 30° of flexion, and a skyline view. The radiographs were classified by an orthopedic knee surgeon using the Kellgren-Lawrence system (Fig. 1), which is an ordinal scale graded from 0 (absence of radiographic features of osteoarthritis) to 4 (large osteophytes, pronounced joint space narrowing, severe sclerosis and defined bone deformity).^9^

### Statistical analysis

Qualitative variables were presented as absolute frequencies and percentages. For quantitative variables, the mean, median, standard deviation, minimum value, and maximum value were calculated. Correlation coefficients between clinical scores and radiographic classification were obtained using Kendall's tau test (τ-Kendall), whose values may vary between -1 and +1. A positive value means that variables have a direct relationship, a negative value means that the variables are inversely related, and zero means there is no correlation between the two variables. The strength of the correlation increases both from 0 to +1, and from 0 to -1. The values of +1 or -1 mean a perfect correlation.^18^

The analysis was performed using the statistical software SPSS v.25 for Windows.

## Results

In total, 120 patients (189 knees) were included in the analysis. The mean age was 64 years (34 to 98 years), with the majority being female (65%). The mean BMI was 30.3 kg/cm^2^. Systemic arterial hypertension was the most common comorbidity (49.1%), followed by diabetes mellitus (16.6%). 57.5% of patients had bilateral gonarthrosis. Epidemiological characteristics and most common comorbidities were described in Table 1.

**Table 1:** Epidemiological characteristics of patients with knee osteoarthritis.

Characteristic	n (%)	Mean (SD)	Median (min‒max)
	n = 120		
*Age (years)*		64.1 (9.5)	64 (34‒98)
*Gender*			
Female	78 (65)		
Male	42 (35)		
*Weight (kg)*		77.9 (15.6)	78 (48‒140)
*Height (cm)*		160.7 (9.2)	160 (142‒180)
*BMI (kg/m^2^)*		30.3 (5.2)	30.1 (20.7‒49.0)
*Smoking*	9 (7.5)		
*Diabetes mellitus*	20 (16.6)		
*Arterial hypertension*	59 (49.1)		
*Dyslipidemia*	11 (9.1)		
*Thyroid disorder*	12 (10)		
*Depression*	5 (4.1)		
*Coronary disease*	5 (4.1)		
*Heart Failure*	10 (8.3)		
*Rheumatoid arthritis*	15 (12.5)		
*Knee osteoarthritis*			
Right	27 (22.5)		
Left	24 (20)		
Bilateral	69 (57.5)		

SD, Standard Deviation; min, minimum value; max, maximum value.

The mean and standard deviation values of the clinical scores according to their distributions in the Kellgren-Lawrence classification are presented in Table 2.

**Table 2:** Mean and standard deviation of clinical scores according to Kellgren-Lawrence classification in TKA candidates.

Clinical score	Kellgren-Lawrence classification		
	Grade 2	Grade 3	Grade 4
	n = 45	n = 62	n=82
**KSS**			
Mean (SD)	42.7 (19.3)	30.7 (20.4)	18.9 (18.4)
**KOOS total**			
Mean (SD)	31.9 (18.2)	22.7 (16.3)	21.5 (16.6)
**KOOS-pain**			
Mean (SD)	35.3 (20.9)	24.7 (18)	24.8 (20)
**KOOS-symptoms**			
Mean (SD)	40 (20.5)	33 (19.7)	29.6 (21.3)
**KOOS-function in daily living**			
Mean (SD)	34.3 (21.2)	23.2 (20)	22.7 (20.9)
**KOOS-sport and recreation function**			
Mean (SD)	12.4 (15.6)	7.2 (12.7)	5.6 (9.4)
**KOOS-quality of life**			
Mean (SD)	23.9 (20.1)	15 (18.8)	13.9 (16.8)

SD, Standard Deviation.

A weak negative correlation was observed between all clinical scores and radiographic classification, and the results were statistically significant under the null hypothesis. The clinical score with the strongest negative correlation was the KSS (τ = -0.356; p < 0.001) and the weakest negative correlation was the KOOS-sport and recreation function (τ = -0.142; p = 0.025). Test results demonstrating correlations between clinical scores and radiographic classification are presented in Table 3.

**Table 3:** Correlation between the KSS and KOOS clinical scores and the Kellgren-Lawrence classification in patients with knee osteoarthritis.

Clinical score	Kellgren-Lawrence classification	p-value
	τ-Kendall	
KSS score objetivo	-0.356	<0.001
KOOS score	-0.166	0.004
KOOS-pain	-0.149	0.010
KOOS-symptoms	-0.159	0.006
KOOS-function in daily living	-0.160	0.005
KOOS-sport and recreation function	-0.142	0.025
KOOS-quality of life	-0.176	0.004

**Fig. 2:**
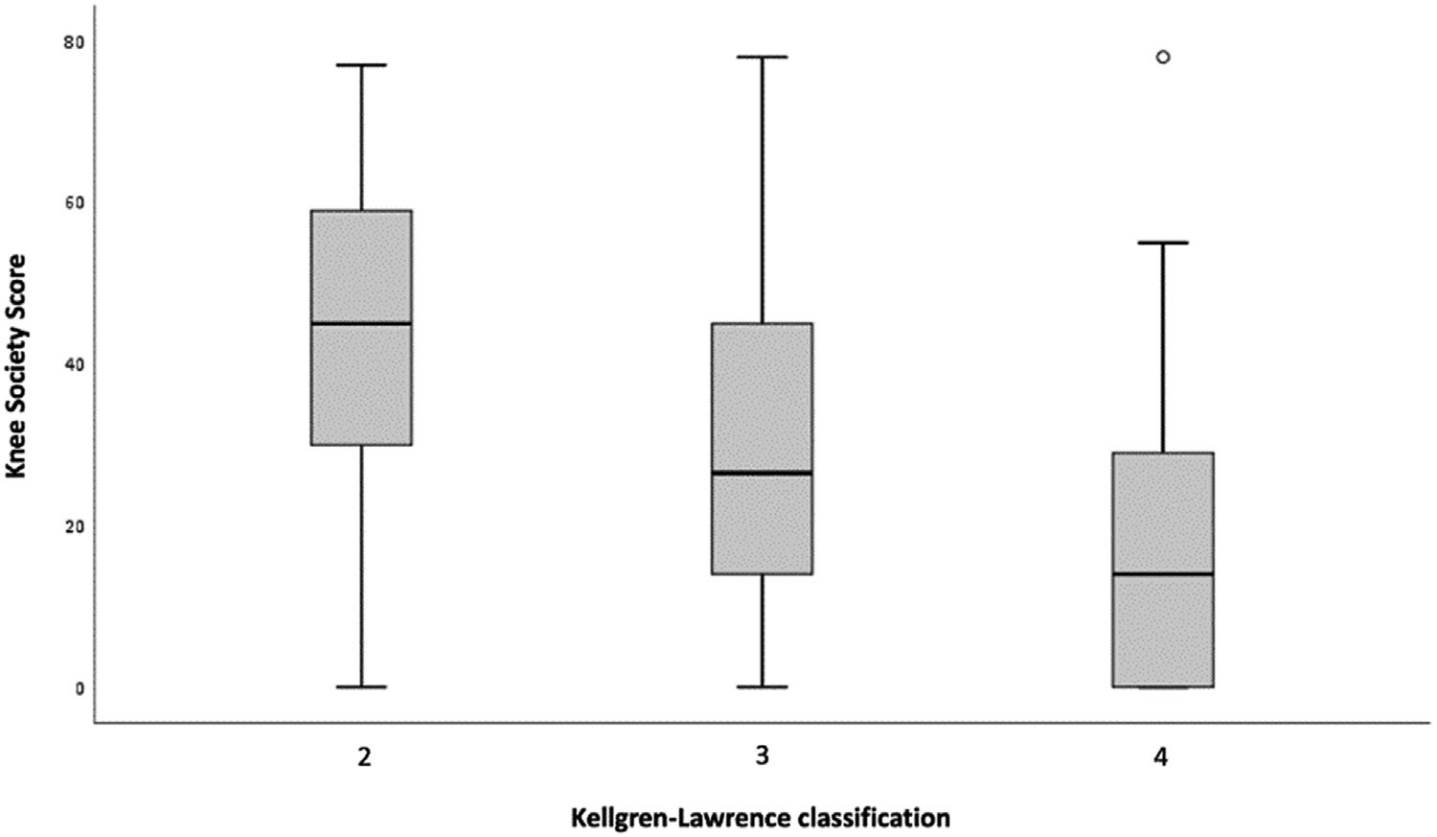
KSS objective score according to the Kellgren-Lawrence classification in patients with knee osteoarthritis.

**Fig. 3:**
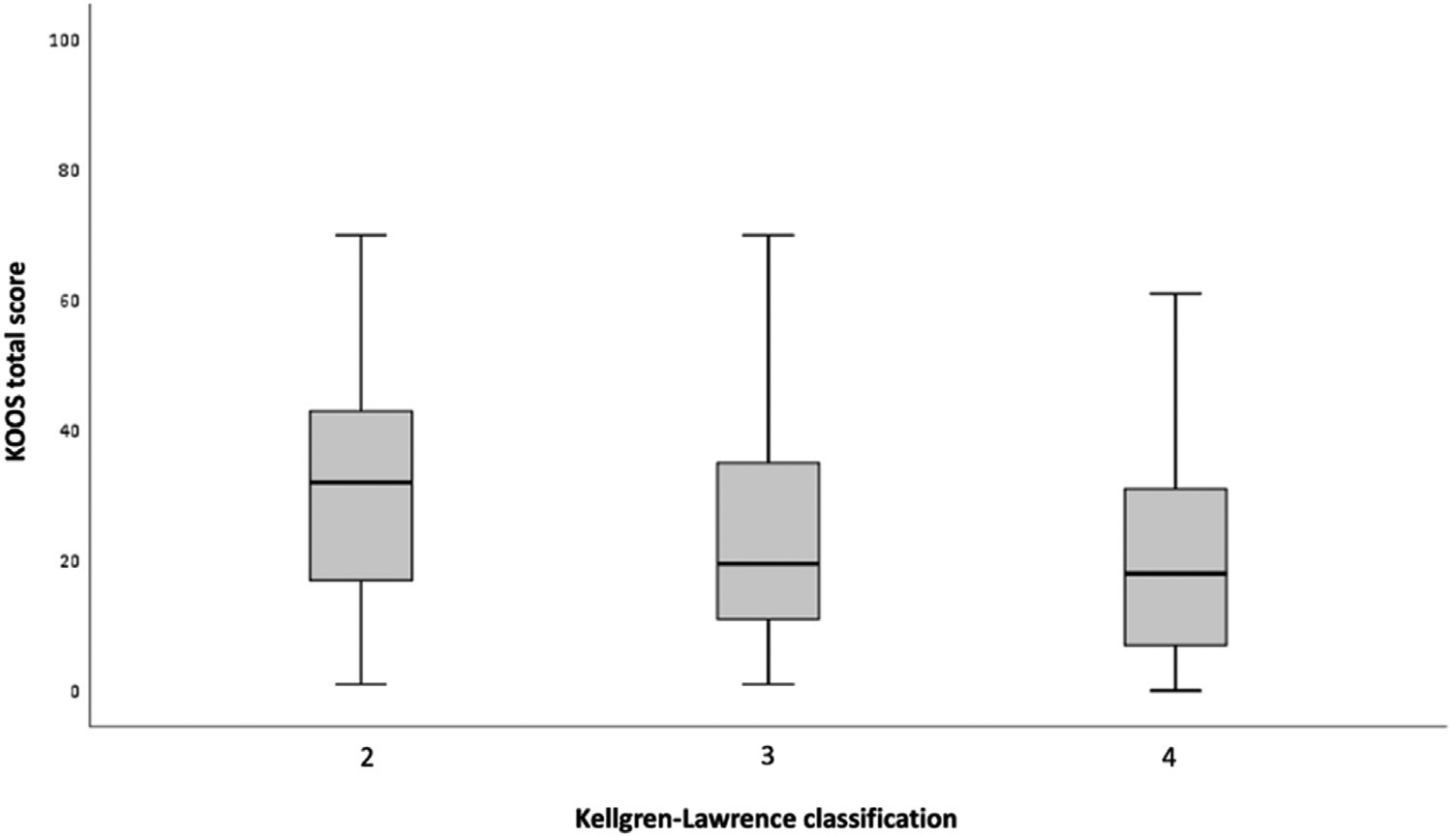
KOOS total score according to the Kellgren-Lawrence classification in patients with knee osteoarthritis.

The distribution of patients between KSS and total KOOS scores according to the radiographic classification are summarized in the following box plots (Figs. 2 and 3)

## Discussion

This study intended to evaluate TKA candidates and correlate their clinical scores with their radiographic classifications, a fact that had not been previously reported in the literature, in addition to carrying out an epidemiological analysis of the population.

In the sample analyzed, the authors found epidemiological data that are similar to those previously described, with a mean age of 64.1 years and a female predominance (65%). In the literature, knee osteoarthritis is found with a frequency of up to 13% in the population aged 60 years or over and is more prevalent in women than in men in the elderly population.^19^ In the present study, obesity proved to be very prevalent, being present in 50% of cases. There is also a high prevalence of systemic arterial hypertension (52.7%) and diabetes mellitus (17.9%). These findings are in accordance with previous literature, in which higher grades in the Ahlbäck radiographic classification^20^ were associated with the occurrence of systemic arterial hypertension, and had a weak correlation with the patient's height and weight, but moderate correlation with BMI.^21^ Furthermore, patients with knee osteoarthritis at Kellgren-Lawrence Grade 2 or above have a higher prevalence of metabolic syndrome, diabetes, and hypertension.^22^

When comparing the mean functional scores reported in this study with those described in the literature, the scores in this cohort were significantly lower, even in the moderate radiographic grades. In a population study that evaluated 981 patients with knee osteoarthritis, a mean total KOOS of 55.33 was found with a standard deviation of ± 20.641.^23^ In this study, patients with Kellgren-Lawrence Grade 2 had a mean total KOOS of 31.87, Grade 3 of 22.65, and Grade 4 of 21.45. These lower clinical score values reported are probably due to the fact that only patients with surgical indications were included in this study, which infers that they have more significant symptoms.

Regarding the clinical-radiographic correlation, a weak and inversely proportional correlation was observed between all clinical scores analyzed, including the subitems, and the Kellgren-Lawrence classification. As described in Table 3, all correlation values between the scores and the radiographic classification were negative, denoting an inversely proportional correlation between these variables, and result values are closer to zero than to -1, denoting a weak correlation. Among clinical scores evaluated, KSS showed the highest correlation with the Kellgren-Lawrence classification (τ = -0.356; p < 0.001), followed by KOOS-quality of life (τ = -0.176; p = 0.004), KOOS-total score (τ=-0.166; p = 0.004), KOOS-function in daily living (τ = -0.160; p = 0.005) and KOOS-symptoms (τ = -0.159; p = 0.006). KOOS-pain (τ = -0.149; p = 0.01) and KOOS-sport and recreation function (τ = -0.142; p = 0.025) scores had the weakest correlations.

Therefore, although radiographic classification is an important parameter in the assessment of patients with knee osteoarthritis, its correlation with the clinical presentation proved to be weak and not very consistent. These findings corroborate previous literature, which shows that the prevalence of pain is greater as radiographic osteoarthritis worsens,^24^ and that these variables present themselves as cause and consequence,^25^ but neither the intensity of pain nor the worsening of other symptoms were consistently associated with radiographic changes.^26^^,^^27^ Other biological, psychological, and social factors probably influence the diverse symptoms of osteoarthrosis and the radiographic worsening is not necessarily decisive in the functional worsening of patients.

Among the limitations of the study are the low reproducibility between observers and the lack of intra-observer uniformity of the Kellgren-Lawrence classification.^28^ Another point is the fact that the Kellgren-Lawrence classification limits the final stage of the disease to Stage 4. However, in the analysis with MRI, a progression of the disease is still seen in patients classified as Stage 4, with worsening of cartilage damage, especially in the less affected tibiofemoral compartment, with fluctuations in bone marrow edema and synovitis. Despite this, Kellgren-Lawrence grades in plain radiographs were highly correlated with the MRI grades.^29^ However, it is noteworthy that the diagnosis of knee osteoarthrosis is more sensitive with the use of MRI than with the Kellgren-Lawrence scale.^30^ As described for the Ahlbäck classification, the Kellgren-Lawrence classification should not be used routinely without the aid of adequate clinical assessment.^31^

## Conclusion

The present study concludes that there is a weak correlation between the severity of radiographic changes and clinical symptoms in candidates for TKA. Among clinical scores, the objective KSS had the highest correlation with radiographic severity.

## Authors’ contributions

Diego Ubrig Munhoz: Conceptualization; methodology; investigation; data curation; writing-original draft.

Andre Giardino Moreira da Silva: Conceptualization; methodology; investigation; data curation; writing-original draft.

Pedro Nogueira Giglio: Methodology; data curation; formal analysis.

Camilo Partezani Helito: Methodology; supervision; project administration.

Riccardo Gomes Gobbi: Conceptualization; supervision; project administration.

Luís Eduardo Passarelli Tirico: Conceptualization; writing-review & editing; supervision; project administration. All authors read and approved the final manuscript.

## Ethics approval

This study was performed in line with the principles of the Declaration of Helsinki. Approval was granted by the Ethics Committee of the University of São Paulo (CAAE 08496919.5.0000.0068; 15/04/2019).

## Declaration of competing interest

The authors declare no conflicts of interest.
